# Indents

**Published:** 1911-08-15

**Authors:** 


### INDENTS.

☞ **Brief Articles of Technical or Scientific Value will receive notice and comment. Contributions invited.**

*Commissioned Status for Army Dental Surgeons.* On March 3, 1911, the bill known as the Army General Appropriation Bill, H. R. 31,237, became law by executive approval.

Hereafter there shall be attached to the Medical Department a dental corps, which shall be composed of dental surgeons and acting dental surgeons, the total number of which shall not exceed the proportion of one to each thousand of actual enlisted strength of the army; the number of dental surgeons shall not exceed sixty, and the number of acting dental surgeons shall be such as may, from time to time, be authorized by law. All original appointments to the dental corps shall be as acting dental surgeons, who shall have the same official status, pay and allowances as the contract dental surgeons now authorized by law. Acting dental surgeons who have served three years in a manner satisfactory to the Secretary of War shall be eligible for appointment as dental surgeons, and, after passing in a satisfactory manner an eximanation which may be prescribed by the Secretary of War, may be commissioned with the rank of first lieutenant in the dental corps to fill the vacancies existing therein. Officers of the dental corps shall have rank in such corps according to date of their commissions therein and shall rank next below officers of the medical reserve corps. Their right to command shall be limited to the dental corps. The pay and allowances of dental surgeons shall be those of first lieutenants, including the right to retirement on account of age or disability, as in the case of other officers: *Provided,* That the time served by dental surgeons as acting dental or contract dental surgeons shall be reckoned in computing the increased service pay of such as are commissioned under this act. The appointees as acting dental surgeons must be citizens of the United States between twenty-one and twenty- seven years of age, graduates of a standard dental college, of good moral character and good professional education, and they shall be required to pass the usual physical exmination required for appointment in the medical corps, and a professional examination which shall include tests of skill in practical dentistry and of proficiency in the usual subjects of a standard dental college course: *Provided,* That the contract dental surgeons attached to the Medical Department at the time of the passage of this act may be eligible for appointment as first lieutenants, dental corps, without limitation as to age: *And provided further,* That the professional examination for such appointment may be waived in the case of contract dental surgeons in the service at the time of the passage of this act whose efficiency reports and entrance examinations are satisfactory. The Secretary of War is authorized to appoint boards of three examiners to conduct the examinations herein prescribed, one of whom shall be a surgeon in the army and two of whom shall be selected by the Secretary of War from the commissioned dental surgeons.

*Occlusion in Bridgework.* I think there is one thing that we all of us are much inclined to neglect in the making of bridge work and that is in the obtaining of correct occlusion in order to obtain the best possible results. I think that all upper posterior bridges should be made to occlude principally upon the lingual cusp in order to secure the greatest strength, and all lower bridges should have the greatest occlusion on the buccal cusp for exactly the same reason. In the case of the upper bridge, we get them the greatest pressure toward the palate bone, which of course gives more rigidly and greater strength than if we were pressing it outward from the center of the circle.—*Dr. Coston in Western Journal.*

*"High-Pressure Anesthesia."* Of a mesio-occlusal cavity in a lower left first molar.

With a No. 3 round bur the clinician drilled into and about one-third of the way through the enamel, immediately outside (bucally) of the line of decay; then with a No. ½ round bur he drilled through another third of the enamel; then used the Myers syringe, and after filling the barrel with a 2 per cent. solution of cocain hydrochlorid placed the point in the above pit, exerting all the pressure possible by squeezing the handles. After about thirty seconds the solution was forced through the remaining third of the enamel and a little into the dentin. After this the No. 3 bur was used until the dentin was reached Then a little pit was made in the dentin by the No. ½ round bur, and the syringe was again used for two minutes, when the tooth was found to be completely desensitized, after which the cavity was prepared by taking in the syringe pit, without any pain whatever, much to the joy and comfort of the patient.—*Dr. Jackman, in Cosmos.*

*"Implantation of An Artificial Root."* An artificial root is made by reinforced wire loops of gold wire, soldered to a disk base having a slot for carrying an artificial tooth or an abutment for a bridge.

This root is constructed of iridio-platinum wire, No. 20 gage, soldered with 24-karat gold. The base, with a groove for the crown attachment, is cast of 22-karat gold, and is soldered to the iridio-platinum frame. This frame is placed into the socket that has been prepared, and is left there for three months before the crown is set. At the end of this time the new growth of bone has filled the socket and holds the root perfectly solid.—*Dr. Greenfield, April Cosmos.*

*Some Precautionary Measures for Casting Under Pressure:* (1) When high-fusing metals such as gold or silver are to be cast, a high heat should be applied to the casting flask. (2) The air and gass should be expelled from the casting flask through the specially provided vents as long as the porosity of the investing material cannot fully absorb them.

(3) Even in case the porosity of the investing material can fully absorb all the air and gas in the casting flask, vents should be provided in the casting flask if the original model to be cast is long and narrow, as in casting the post of an artificial crown.

(4) The vents should be arranged in a line running upward diagonally from one corner of the casting flask and ending near the sprue.

(5) With heavy metals, notably high-fusing metals such as gold and silver, the heat should be raised high above their fusing-points before forcing them into the mold cavity.

(6) In casting such metals, the sprue hole, if it be large, should be covered till the mass of metal is completely molten.

(7) If a molten light metal is seen running out through the vent tube on being pressed in, it is necessary either to block up the mouth of the tube or to cool it so that the molten metal will congeal while the pressure is being exerted.

(8) The porcelain teeth now in vogue being impe fect for casting purposes, it is necessary that teeth exclusively for casting purposes should be produced.

(9) Every cast metal, no matter what its nature, should be annealed.—*Dr. Enomoto, in April Cosmos.*

*Porcelain Crown— Cast Gold Base.* Select a pinless crown (any make) to suit case. I find the Dentsply Crowns preferable in most cases. Trim the root a little beyond gum line, all around. Fit a roughened iridio platinum wire, 14 to 16 gauge, to canal, extending beyond root, to length of hole in crown. Grind crown to contact with root at labial, but make it short toward lingual, leveling edge all around except at labial, so that wax (and gold) base will be somewhat cup-shaped. Oil base of crown, soften small piece of wax. Heat pin and melt into soft wax; then press pin and soft wax into hole and against base of crown, and force to place upon root; trim, cool, and remove. Carve wax carefully, adding a little if necessary, and place once more upon root. Remove carefully and invest root end of pin and wax with crown in place. Later remove crown, attach sprue, soak thoroughly and finish investing. After the investment has thoroughly set, been dried out gradually, and heated almost to a red heat, cast with pure gold at a white heat. Adjust to crown and root, polish, and set to both with one mix of cement.

Dentsply diatoric bicuspids make good crowns with gold base, as above, after filling lateral holes and undercuts with gutta percha or cement and gold foil. Or with gold cast directly on to the porcelain and into the entire retentive form of the tooth. The porcelain should be at a red heat when the gold is cast, and of course, be allowed to cool gradually. I have made and set quite a number of these crowns, only one of which has failed, in which case I simply selected a new tooth of same mould, ground off lateral arms of gold base remaining upon root, and cemented new tooth to place.—V. C. S. in *Digest.*

*Filling Cervical Cavities.* Cervical cavities, when they run clear around a molar— from the buccal surface around to the distal and sometimes lingually—how he is going to fill that with gold? I fill them with amalgam. I have to use amalgam once in a while. I would not use, in filling these cervical surfaces, a rubber dam—a cruel procedure, in my opinion. I do not see any necessity for it so long as we can use the gold inlay, and it is not necessary to use the cervical clamp when you are inserting a gold inlay.

In many of these surfaces I speak about I do not see how they can be filled save with amalgam. The essayist says they cannot be filled with amalgam because you press the material in at one point and it will come out at the other. Very true. Do not do it that way. You have, we will say, in a third molar a cavity which runs around the back of the tooth; you prepare your cavity; in the first place, fill half of it one day, and let it go a while; then make another cavity of the remaining portion—I have made three different cavities out of these cervical fissures, running all around a tooth. How else can you do it, except you make three inlays? I think, therefore, amalgam in those cases is the best material we have to resort to, and if such fillings are properly finished you can make them, by the use of proper files, as smooth as the gold.

There are certain cases were we have to use amalgam, and I am glad to do it. The only objection to the material is its unseemly color, and we all dislike it on that acc unt. It is not necessary, in putting in amalgam, to have the cavity perfectly dry. The amalgam forced into the cavity will expel the moisture. If you use the copper amalgam, it does not matter whether the cavity be dry or not.—Dr. Patterson in *Western Journal.*

*To obtain clean results in Casting Inlays and Bridges.* This depends not only on a well-finished wax model, the surface of which has been smoothed over with vaseline, and this in turn removed by means of alcohol, but also on the manner in which the same has been invested and heated. I have obtained my best results by first painting the wax model, after mounting it on the sprue wire, with soapy water, just prior to investing, and then applying the investment material over this with a camel's hair brush. The soapy water causes the investment to flow evenly over, and adhere to the wax model. The investment should be thoroughly mixed for a few minutes to a creamy consistency, and then by rolling the mixing bowl slowly round on its side all air bubbles are excluded. In heating, do not bring the ring in direct contact with the flame, but place a thin sheet of asbestos in between, which obviates any liability as to the investment cracking.—A. Dangar Burne, Sydney, N. S. W., Australia, *Pacific Dental Gazette.*

*A New Local Anaesthetic.* In some eighteen cases, including embedded third molars, which are the most trying for both patient and operator, I have accomplished an absolutely painless operation with the following formula, with an anaesthesia lasting from two to twelve hours:

℞—Novocain and Adrenalin (P. D. Tablets 1-3 gr. Novocain 1-200 Adrenalin)
Sodii Bicarb. Sol. ($\frac{1}{8}$ gr. to the minim.)
Normal Saline Sol. 3 c.c.

The absolute safety of this solution recommends itself at a glance over any cocain product, with the added advantage of a freshly prepared solution for each operation. The sodium bicarbonate solution acts on the nerve terminals and thus prolongs the anaesthesia.

I have my druggist prepare the sodium bicarbonate solution so that each minim represents $\frac{1}{8}$ gr. Using a minim dropper the tablet is dissolved in a graduate in the bicarbonate solution, adding 3c.c. of the normal saline solution.

The usual procedure for local anaesthsia is followed, injecting around the end of the roots. I usually wait from five to eight minutes before operating, as the anaesthesia deepens.

The after pain so often experienced with cocain solution is eliminated, as is the soreness consequent to the operation, for by the time the anaesthesia wears off the shock has passed away entirely.

I would recommend this formula for all surgical operations of the mouth where a local anaesthetic is to be used. —Dr. J. O. Reinstein in *Brief.*

*A Bridge Denture Swallowed.* The patient, a female, aged 36, had an upper bridge, composed of five teeth, as follows: Left upper cuspid, a Richmond crown; the first upper left molar, a gold crown; these served as abutments for two left upper bicuspids and the second left upper molar. The above bridge was inserted in August 1908. I lost track of the patient until the 21st of December, 1910, when I was called to see her about 7 A. M. This is the history.

The patient had oatmeal for breakfast, and during an act of swallowing felt the above described bridge pass into the pharynx and esophagus. She claimed that the bridge was loose for about ten days.

The patient was very hysterical. For treatment I gave, first, a hypodermic of morphine gr. $\frac{1}{8}$ and atropine gr. 1-120 to stop the pain experienced along the course of the esophagus. This was followed by a cup of hot, black coffee, to counteract the rapid, wiry, compressible pulse with symptoms of "shock."

The following diet was advised: mashed potatoes, hard boiled eggs, oatmeal, without milk; rice and calf's foot jelly; the object of this dietary being to agglutinate the bridge so that the platinum pin of the Richmond crown should not injure the gastro-intestinal tract, especially the appendical region.

On the 22d of December I called again and found the patient in a fairly good condition and without pain or discomfort. I prescribed the following:

℞—Tinc. nucis vomicae ............................ ʒiss

Tinc. belladonnae ............................ ʒi

Bismuthi subnitratis ............................ ʒiii

Syrupi rhei aromatici ............................ q.s. ℥ii

.M. Sig.—ʒi followed by water every 2 hours. Shake before using.

I ordered a continuance of the agglutinative diet with a more liberal liquid allowance. My instructions were not to use purgatives of any kind, and to examine all fecal movements.

On December 23 I was informed that the patient had had a bowel movement, but minus the bridge.

I then ordered the following:

℞—Fluid extract cascara ............................ ʒi

Mucilage acaciae ............................ q.s. ℥ii

M. Sig.—ʒi three times daily.

On December 24, at 10 A. M., I was informed by phone that the patient had had a bowel movement plus the bridge. The bridge was agglutinated and the pin was entirely covered, and the patient, so far as I could determine, in perfect condition.

I shall be glad to receive from those interested criticism of the treatment given in this case, and also data regarding similar cases in the practice of others.—Wm. Samueli, D.D.S. in *Brief*.

*To make solid Gold Dummies.* In constructing a bridge where the teeth are out of sight, or where you want to make an all-gold bridge, a very nice and accurate way to make the dummies is to select suitable teeth for the case and articulate them, then use these same teeth from which make your moulds. To make the moulds, set the teeth in mouldine pins down, leaving the buccal and grinding surfaces exposed; set your rubber ring over them; melt your fusible metal and pour it in; after the metal cools, remove the tooth, and you have a nice mould in which to make your inlay-wax model; now cast your tooth from this wax model the same as you would cast an inlay.—Dr. E. T. McKim, Shell City, Mo. in *Summary*.

